# Feasibility Analysis on the Use of Ultrasonic Communications for Body Sensor Networks

**DOI:** 10.3390/s18124496

**Published:** 2018-12-19

**Authors:** Meina Li, Youn Tae Kim

**Affiliations:** 1 College of Instrumentation and Electrical Engineering, Jilin University, Changchun 130061, China; 2 Departement of IT Fusion Technology, Graduate School, Chosun University, Gwangju 61452, Korea

**Keywords:** ultrasonic communication, body sensor network (BSN), healthcare, wireless communication

## Abstract

Ultrasonic waves have good propagation in the human body and have been widely applied in biomedical device design without any reported side effects. Therefore, ultrasonic waves can provide an alternative method as an information carrier for body sensor networks (BSNs). This paper presents a novel wireless communication method that uses ultrasonic sound waves as a medium for healthcare systems. We investigated the feasibility of our proposal by testing it in a real digital communication experimental setup. To find an acceptable modulation method, the functionality of the proposed ultrasound-based digital communication approach was tested involving three principal modulation methods: amplitude shift keying (ASK), frequency shift keying (FSK), and phase shift keying (PSK). The modulated digital signals obtained from the experiments were compared with the simulated signals. Analysis of the results shows that ultrasonic waves are feasible and can be used for digital communication.

## 1. Introduction

With the increasing attention being paid to healthcare, wireless sensor networks for physiological monitoring have been widely applied in daily life. The rapid development of biosensors provides a means for real-time monitoring for healthcare and can help realize ubiquitous healthcare. Patients could be monitored by doctors from a personal monitoring device in any environment and without restricting activity. Nanoscale sensors can detect physiological parameters inside the body and then transmit signals to a sensor attached on the skin. Thereafter, the data can be wirelessly transmitted by radio frequency (RF) communication networks. Various body sensors, such as accelerometers and monitors for ECG, temperature, glucose, and SpO_2_, have been developed for pervasive healthcare monitoring systems [1]. These sensors are placed inside the body or attached to the skin to monitor the health status of a patient. For example, diabetics use an implanted glucose sensor and a wearable insulin actuator to adjust their glucose level. When the glucose level increases, the glucose sensor transmits a signal to the actuator, which has a microneedle attached to the skin that triggers the injection of insulin. The system monitors the patient in real-time, and does not require patient hospitalization. Physiological data can be wirelessly transmitted through RF to doctors for analysis [2]. This can reduce the patient healthcare costs and the frequency of hospital visits. With recent developments in nanotechnology, materials science, and integrated circuits, researchers are focusing more attention on the demands of wearable healthcare systems. However, there are still few standards or effective communication methods for body-area networks, especially for implanted sensors. Therefore, communication among sensor nodes is an important role for current and future device development.

RF waves have been successfully used in wireless communication systems. Conventionally, in body sensor networks (BSNs), an RF microchip is embedded in the sensor board for communication [3,4]. To reduce the size of the sensor module, it is necessary to reduce the size of the RF antenna chips [5,6,7]. The RF modules in portable devices also consume battery power quickly [8,9]. This is a concern for implantable sensors, because it is difficult to change the batteries frequently. Depending on the specific environment, RF electromagnetic waves have poor propagation in water. This is a particular problem for in-body communication, because 60% to 70% of the human body is made of water. The RF signals transmitted through tissues suffer high attenuation. To enhance the signal, high-power RF devices are used, which can result in heating of soft tissue because of absorption. Furthermore, RF signals are easily affected when other electronic devices operate at the same frequency. The RF bandwidth is limited, which inherently limits the communication rates [10]. Physical monitoring data are considered confidential personal medical information, which requires the wireless system to be secure to prevent eavesdropping or interference. A lack of security could be a risk for the patient, especially in the case of a monitoring board with a therapy actuator. Prolonged RF radiation can overheat tissue, which can lead to bioeffects [11,12]. Health concerns therefore impose restrictions on RF signal levels.

To address all of these issues, we propose using ultrasonic waves as the communication medium for healthcare systems. Ultrasound has been pervasively applied in medicine [13]. Therefore, it can be an alternative method for short-distance digital communication. Ultrasound has advantages over electromagnetic waves in the application of implantable medical devices: The human body is composed mostly of water, and RF waves are subject to high absorption. For communication purposes, an RF module requires high transmission power. That requirement increases the size and weight of an implantable sensor. Researchers have thus focused on microsize and low power consumption for implantable sensors. Furthermore, the RF spectrum hosts many devices, such as Wi-Fi and Bluetooth devices. The interference generated can affect the reliability of intrabody networks and the security of patients. Information about patients could be eavesdropped upon, which causes a security concern. Ultrasound could propagate along the body or inside the body, which enhances security. In addition, the frequency choice is more flexible compared with that of RF communication. From research conducted over the past decades, ultrasonic waves are known to have better propagation characteristics than RF waves in medical applications. Previous studies have provided software-based ultrasonic sensor networks; however, there was no real experimental realization [14,15,16] on digital communication. Therefore, we attempted to use ultrasonic wave communication between sensors on the basis of an experimental setup used for body sensor networks.

The rest of this paper is organized as follows. In Section 2, we introduce the principles of ultrasonic waves, including the attenuation of ultrasonic waves and the fundamentals of propagation along the body. In Section 3, we illustrate the modulation method of communication and the design of the experimental setup, and discuss the experimental and simulation waveforms. In Section 4, we present the results and discuss the performance of ultrasonic waves through a BSN. The conclusion is provided in Section 5.

## 2. Principles of Ultrasonic Waves

To use ultrasonic waves for communication, understanding the physical characteristics of ultrasonic waves is necessary. Ultrasonic waves are mechanical waves; the signal attenuation is proportional to the distance and depends on the propagation medium. As a result of this, ultrasonic waves are suitable for short-range communication. From our research, we list three major principles of ultrasonic waves that can cause energy dissipation through the transmission.

### 2.1. Attenuation

Attenuation can be defined as the loss of a signal’s amplitude with increasing propagation distance. Contributions to the attenuation of an ultrasound beam may include absorption, scattering, reflection, refraction, diffraction, interference, and divergence. Among these factors, two major classes of attenuation should be considered in ultrasonic materials characterization. The first is absorption; the absorbed energy is irreversibly lost from transmission through the medium. The lost energy is primarily transformed into heat. The second is scattering; this appears in non-uniform media. In our research, only one medium was considered. Scattering is not the main energy dissipation in our research. The attenuation can be described as

Attenuation= *α* [dB/(MHz·cm)]·*l* [cm]·*f*[MHz], (1)

where *α* is the attenuation coefficient, *f* is the fundamental frequency of the ultrasonic wave, and *l* is the distance from the source point. From experiments, spread and far-field effects cause much more energy dissipation, consistent with theory and experimental results.

### 2.2. Spread

Ultrasound waves can be both emitted and received by a transducer. Most ultrasonic transducers are of finite diameter. Therefore, sound energy is lost as a result of beam expansion. Spreading loss occurs because the total amount of energy in a wave remains the same as it spreads out from a source. When the wave spread is larger, it needs the energy to fill it. Therefore, the far distances get small energy. Attenuation from a point source, where the intensity decreases according to the square of the distance from the source, is shown by [17]:

(2) $$\Delta D=10\log{\left(\frac{d_{1}}{d_{2}}\right)}^{2}=20\log\left(\frac{d_{1}}{d_{2}}\right),$$

where ΔD is the energy reduction in decibels (dB), and at the *d* distance from the source point. Under ideal conditions, a sound level drops 6 dB for every doubling of the distance from the source. This formula was demonstrated by our experimental environment setup test. The experimental results are consistent with those of the theory.

### 2.3. Far Field

**Figure 1:**
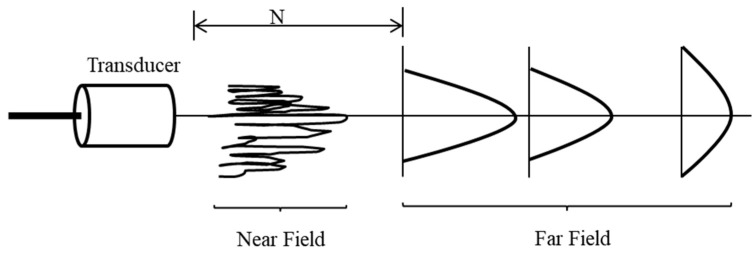
Beam profile with different distances.

The characterizations of ultrasonic beams are classified by two areas, near field and far field, as shown in Figure 1. The amplitude in the near field is irregular and can be extremely difficult to accurately evaluate in transmission. The area beyond the near field, where the amplitude of the ultrasonic beam is more uniform, is called the far field. In this area, the amplitude becomes inversely proportional to the distance from the transducer.

The far field is the area beyond N where the sound field pressure gradually drops to zero. Due to the variations within the near field, it can be difficult to accurately evaluate flaws using amplitude. In the experimental design, the receiver should be placed in the far field. The distance starting from the far field can be calculated by Equation (3), where *D* is the diameter of the ultrasound transducer and λ is the wavelength in the transmission medium.

(3) $$N>\frac{D^{2}}{4\lambda }$$

## 3. Method

**Figure 2:**
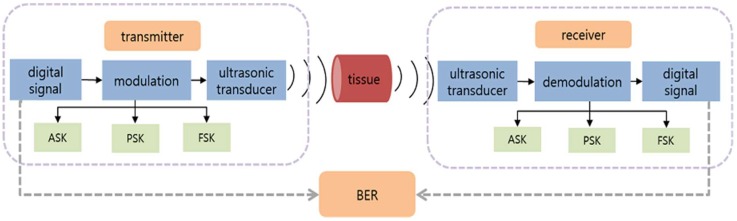
Architecture of ultrasonic communication for body sensor networks.

The ultrasonic communication system was designed in two parts. The transmission part includes the signal generator which generates digital signal, modulation, and ultrasonic transducer. Three principal digital communication modulation schemes were used in the experiments: amplitude shift keying (ASK), frequency shift keying (FSK), and phase shift keying (PSK). In this study, only binary modulation was considered. Binary ASK is in the term of on-off keying (OOK). The receiver part included the ultrasonic transducer as the receiver. The digital signal was modulated and then sent to the ultrasonic transducer. The received ultrasound signal was demodulated to the original digital signal. Finally, the bit error rate (BER) was calculated for evaluation of the three modulation methods. Figure 2 shows the architecture of the ultrasonic communication for BSNs.

ASK is a form of amplitude modulation that represents digital signals as amplitude variations of the carrier wave. Binary ASK signaling is the simplest form of ASK with two symbol states. A zero-amplitude carrier transmission represents a 0, and a non-zero amplitude indicates a 1. Binary ASK signaling is also OOK. The general analytical expression for the modulated signal *S_i_*(*t*) is

(4) $$S_{i}\left(t\right)=\;\sqrt{\frac{2E_{i}\left(t\right)}{T}}\cos\left(\omega_{0}t+\;\varphi \right)t\;\leq \;T;\;i\;=\;1,\ldots ,M,$$

where ***E*** is the symbol energy, *T* is the symbol time duration, the amplitude term $\sqrt{2E_{i}\left(t\right)/T}$ has *M* discrete values, and *φ* is the phase. In the case of OOK, *M* is equal to 2, corresponding to two waveform types.

FSK is a frequency modulation scheme that conveys data using distinct frequencies to represent symbol states. The simplest FSK is binary FSK (BFSK). BFSK uses a pair of discrete frequencies to transmit binary 0 and 1 information. The general analytical expression for a modulated BFSK signal is

(5) $$S_{i}\left(t\right)=\;\sqrt{\frac{2E}{T}}\cos\left(\omega_{i}t+\;\varphi \right)\;0\;\leq \;t\;\leq \;T;\;i\;=\;1,\ldots ,M.$$

PSK conveys data by changing the phase of the carrier. Binary PSK (BPSK) here has only two phases, 0 and π. The mathematical expression is

(6) $$\;S_{i}\left(t\right)=\;\sqrt{\frac{2E}{T}}\cos\left(\omega_{0}t+\varphi_{i}\left(t\right)\right)\;0\leq t\leq T;\;i=1,\ldots ,M.$$

### 3.1. Experimental Setup

**Figure 3:**
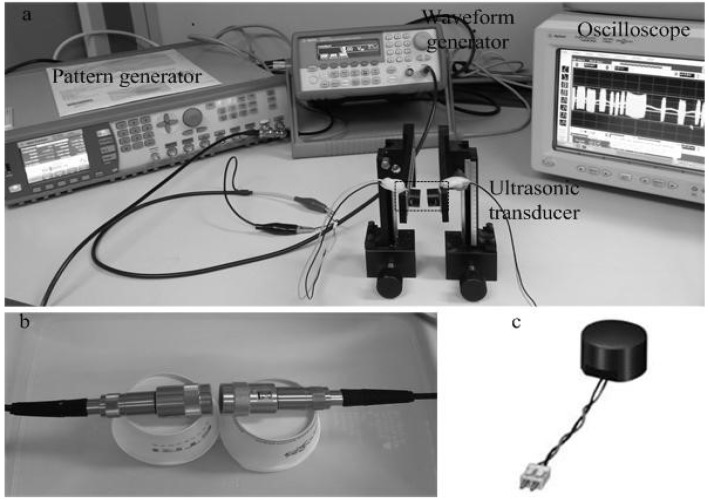
(**a**) Experimental setup; (**b**) Transducer for frequency shift keying (FSK) modulation; (**c**) Transducer for amplitude shift keying (ASK), and phase shift keying (PSK) modulation.

To set up the experiment, we considered several points. First, we could not use a human subject for the experiment because of ethical considerations. Hence, beef was considered as a substitute for soft tissue. Second, ultrasonic transducers emit ultrasonic waves in a propagation medium such asin air or water environment. Third, the narrow frequency bandwidth of the ultrasonic transducer is a limitation, especially for FSK modulation. Fourth, the different healthcare communication scenarios, such as in-body to in-body, in-body to on-body, and on-body to off-body, had to be considered. For BSNs, communication could be inside the body. With one sensor on the skin and the other under the skin, the inside sensor could monitor physical parameters, such as blood inside the body, then transmit the information to the receiver on the skin. Next, the sensor on the skin transmits the signal to the hospital via an RF communication method. The distance between the two sensors measures from a few mm up to 1 cm, considered applicable for short-range communications. Therefore, we do not consider long distances for inside body communication. The application is applicable for implantable sensors because it is designed for short-range communications. The experimental setup was operated in air and water environments based on the property of two types of ultrasonic transducers, as shown in Figure 3. The commercial ultrasonic transducer has limited frequencies. We first chose the Murata (40 kHz) transducer for OOK and BPSK modulation. However, it could not receive signals for BFSK modulation because of its narrow bandwidth. Next, we chose the Olympus 500 kHz for BFSK modulation, which is a relatively low frequency commercial ultrasonic transducer. With an increasing operating frequency, the size of the transducer is decreased, resulting in higher signal attenuation [2,18]. It should be considered among frequency and attenuation. Owing to the limited frequencies of the commercial ultrasonic transducer, we did not have many tradeoffs for these factors during the experiments. The tradeoffs among these factors will drive the design for a specific application.

Binary bits were generated by a pattern generator. A waveform generator was used to generate ASK, FSK, and PSK signals. The output ultrasonic signals were measured by an oscilloscope. Figure 3b shows the ultrasonic transducer (Olympus) that was used in water for FSK modulation [19]. The transducer shown in Figure 3c (Murata) was used in air for ASK and PSK modulation [20].

### 3.2. OOK Modulation

**Figure 4:**
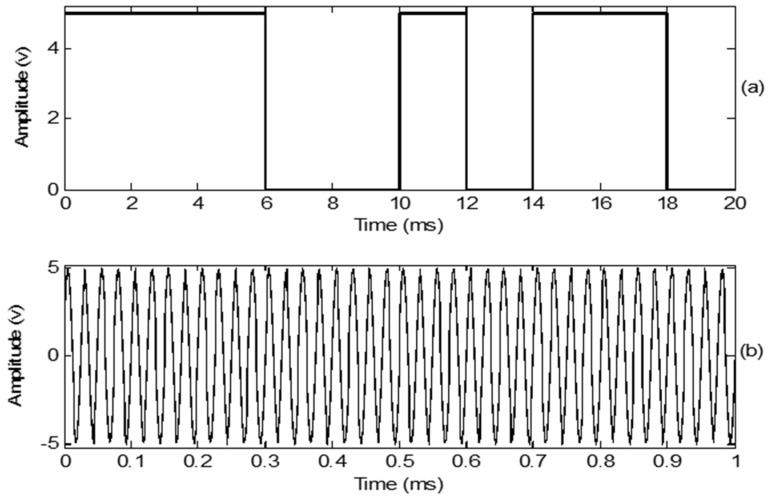
(**a**) Baseband digital signal at 500 bps; (**b**) Carrier signal at 40 kHz.

The input digital signal was a 10 bit binary stream (1110010110) at a data rate of 500 bps. The ultrasonic transducer frequency was 40 kHz for OOK and BPSK modulation. The amplitude was set to 5 V. The baseband digital and carrier signals are shown in Figure 4.

**Figure 5:**
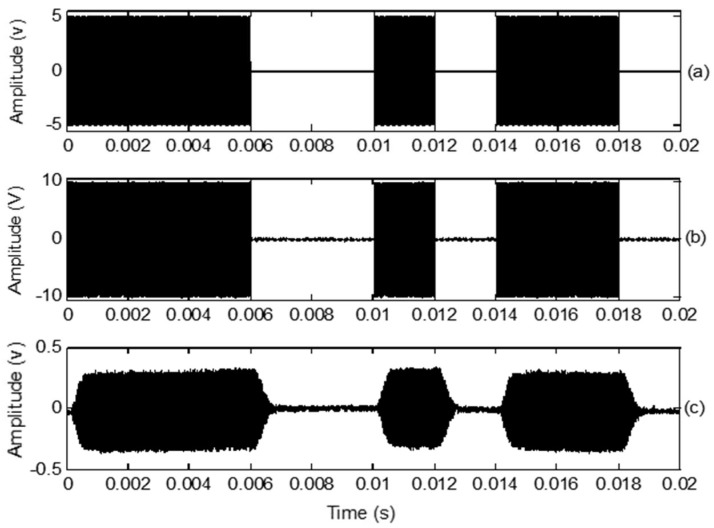
(**a**) Simulation of on-off keying (OOK) signals with 40 kHz carrier frequencies; (**b**) Transmitted OOK signal; (**c**) Received OOK signal in air environment.

The simulated, transmitted, and received OOK signals are shown in Figure 5. The carrier frequency was set to 40 kHz. The simulated OOK signal was theoretically calculated in MATLAB, as shown in Figure 5a. Figure 5b shows the oscilloscope trace of the transmitted OOK signals generated without connecting the ultrasonic transducer. Figure 5c shows that the amplitude of the received OOK signals is sharply reduced; however, the symbol sequences are still distinguishable. The transition from 1 to 0 or 0 to 1 has a delay, which is caused by the impulse response of the transmitting and receiving transducers. At high data rates, the waveform was highly distorted.

### 3.3. BPSK Modulation

**Figure 6:**
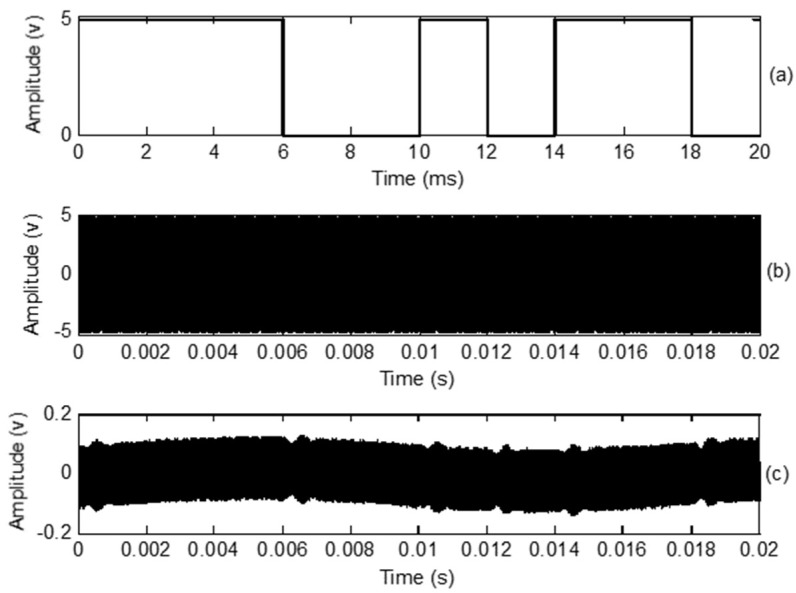
(**a**) Baseband digital signal at 500 bps; (**b**) Transmitted BPSK signal; (**c**) Received BPSK signal.

**Figure 7:**
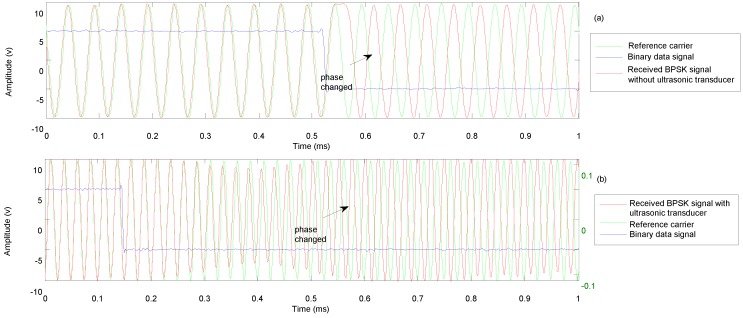
(**a**) Transition of transmittedbinary PSK (BPSK) signal; (**b**) Transition of received BPSK signal.

Figure 6 shows the baseband digital signal along with the transmitted and received BPSK signals. The carrier frequency was chosen as 40 kHz. A 180° phase change was imposed when the symbol changed from 1 to 0 or 0 to 1. As shown in Figure 6c, there was a small “bump” when the symbol changed. The shape of the received BPSK signal was sinusoidal. Figure 7 is an enlargement of the 1 to 0 transition in the transmitted (Figure 7a) and received signals (Figure 7b). It can be seen that the phase of the transmitted signal rapidly changes with the digital signal. The received signal is obtained after the transmitted signal passes through the propagation medium and both ultrasonic transducers. In Figure 7b, it can be seen that the received signal remains in-phase with the reference carrier for some time, and the phase then changes along with an amplitude reduction. Finally, the amplitude recovers when a phase change of 180° is achieved. This causes the “bump” in the received signal.

### 3.4. BFSK Modulation

**Figure 8:**
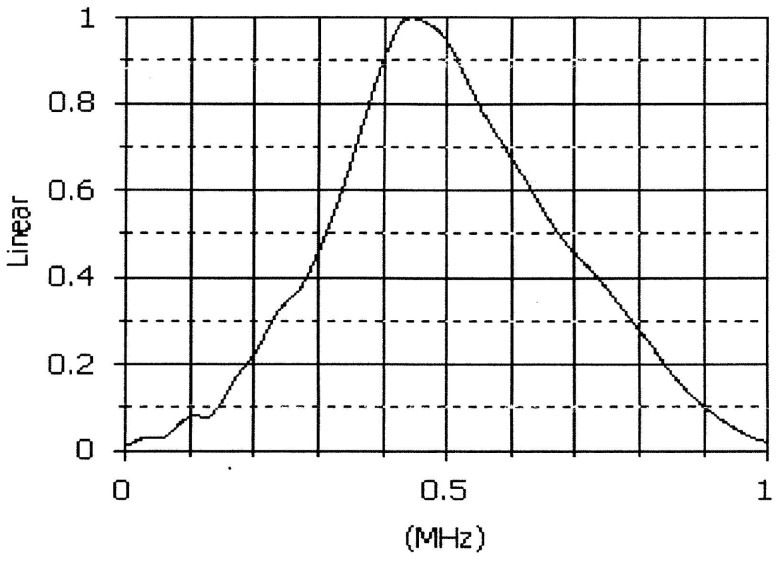
Frequency spectrum of the ultrasonic transducer.

Two discrete frequencies are required for BFSK. According to our survey, almost all commercial ultrasonic transducers have a narrow bandwidth; empirical tests had to be carried out to obtain the same amplitude at different frequencies. We used 500 kHz central frequency ultrasonic transducers (Olympus Immersion Transducer V318) in our experiment. The transducer response to frequency is shown in Figure 8. The test was performed five times and the average voltage was recorded. The amplitude test results are shown in Table 1. The input amplitude is 5 V. The 470 and 490 kHz frequencies have similar amplitudes, as indicated by the bold characters. Finally, 470 and 490 kHz were selected to represent 0 and 1, respectively.

**Figure 9:**
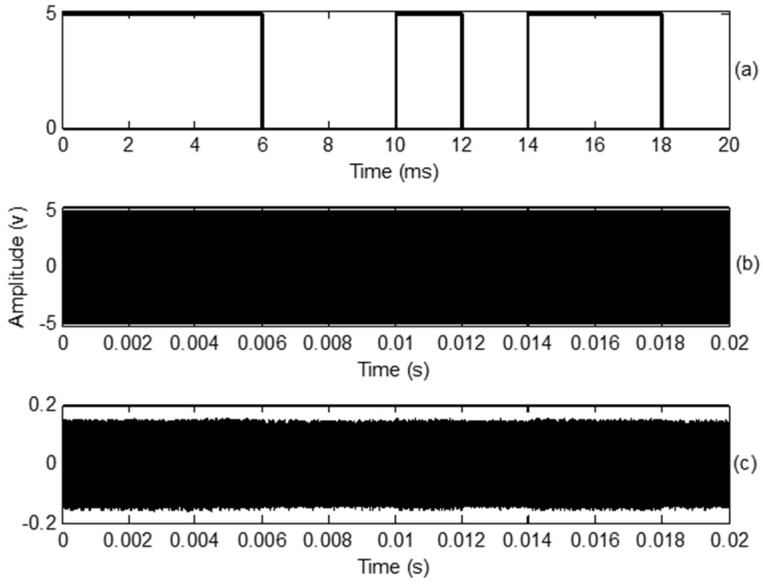
(**a**) Baseband digital signal at 500 bps; (**b**) Transmitted BFSK signal; (**c**) Received BFSK signal in water environment.

BFSK modulation was used for the experiment in water. The baseband digital signal, transmitted BFSK signal, and received BFSK signal are compared in Figure 9. The received BFSK signal is not as severely delayed as the OOK and BPSK signals. The data rate and shape were also not affected much. The frequency shift was examined with an oscilloscope. The shift time depended on the data rate, as in the other two modulation schemes.

## 4. Results

**Figure 10:**
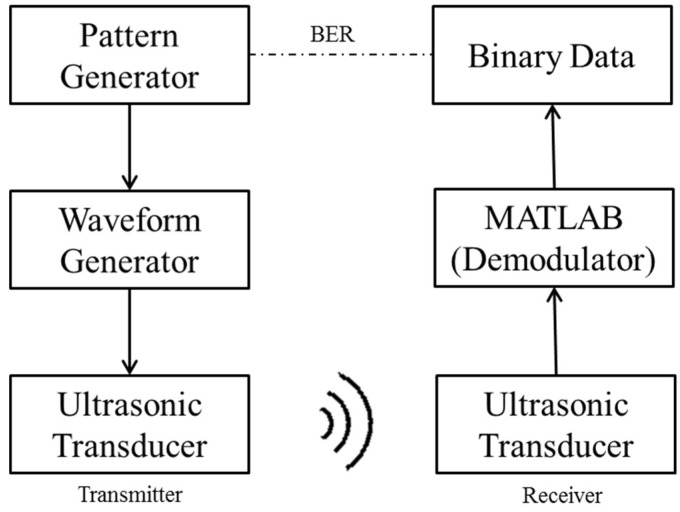
Workflow of signal processing.

**Figure 11:**
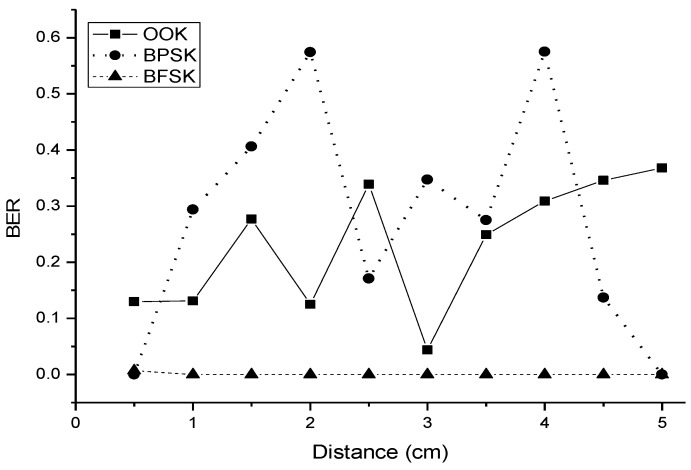
Bit error rate over different transmission ranges for the three modulation schemes in the experimental results.

The received signals were demodulated by MATLAB. The demodulation depends on the signal energy, and the threshold value for making decisions for each symbol was set by trial and error. The processing is shown in Figure 10. The BER was calculated in increments of 0.5 cm from 0.5 to 5 cm, as shown in Figure 11. Owing to the data storage limitations of the oscilloscope, the BFSK experiment was limited to a 100 bit stream, and the OOK and BPSK modulation experiments were limited to a 1000 bit stream. Over the three demodulation schemes, BFSK had the lowest BER. This indicates that an ultrasonic wave has better transmission in water than in air. Therefore, we could use the transducer inside the body. The BER for BFSK modulation remains the same as the distance increases. The stability of the BER for BFSK modulation results from the smaller binary stream storage used in the experiment. OOK and BPSK modulation schemes have a small BER for short distances, and then the trend becomes irregular. When ultrasonic waves propagate in air or water, the energy of the received signal is attenuated as the distance increases. Even though the results do not exhibit any increasing trend with distance, the BERs of the three modulation schemes are acceptable.

## 5. Conclusions and Future Work

The novelty of this study was in its use of ultrasonic waves for wireless communication for healthcare systems. The experimental results for the three principal modulation schemes of OOK, BPSK, and BFSK are provided. The experiment was set up in both water and air. The binary data rate was set to 500 bps, and the carrier frequencies were 40 kHz for OOK and BPSK, and 470 kHz and 480 kHz for BFSK modulation. Although all three modulation schemes show potential for digital communication, BFSK had better performance in terms of both the BER and the shape of the modulation signal. OOK had a higher average voltage (0.3 V) than BFSK (0.18 V) and BPSK (0.15 V).

This study is based on an analysis of the feasibility of using ultrasonic communication. It is difficult to detect the signal from the receiver. To detect the signal, we studied the physical characteristics of ultrasound, frequency selection; investigated commercial transducers and their properties for analysis, and designed the experiment. In a future study, we will attempt to use an amplifier, apply robust modulation, and enhance the demodulation algorithm to reduce the BER.

## Figures and Tables

**Table 1:** Ultrasonic transducer amplitude test for the frequency selection.

Frequency (kHz)	Amplitude (mV)
460	285.8
**470**	**280.5**
480	283.0
**490**	**279.3**
500	264.3
510	225.1
